# Effects of High-Frequency (HF) Repetitive Transcranial Magnetic Stimulation (rTMS) on Upper Extremity Motor Function in Stroke Patients: A Systematic Review

**DOI:** 10.3390/medicina57111215

**Published:** 2021-11-07

**Authors:** Birute Vabalaite, Laura Petruseviciene, Raimondas Savickas, Raimondas Kubilius, Povilas Ignatavicius, Egle Lendraitiene

**Affiliations:** 1 Department of Rehabilitation, Hospital of Lithuanian University of Health Sciences Kauno Klinikos, Eiveniu Str. 2, LT-50161 Kaunas, Lithuania; lciginskaite@gmail.com (L.P.); raimondas.savickas@kaunoklinikos.lt (R.S.); raimondas.kubilius@kaunoklinikos.lt (R.K.); egle.lendraitiene@kaunoklinikos.lt (E.L.); 2 Department of Rehabilitation, Lithuanian University of Health Sciences, Mickeviciaus Str. 7, LT-44307 Kaunas, Lithuania; 3 Department of Surgery, Hospital of Lithuanian University of Health Sciences Kaunas Klinikos, Eiveniu Str. 2, LT-50161 Kaunas, Lithuania; povilas.ignatavicius@kaunoklinikos.lt

**Keywords:** neurological rehabilitation, transcranial magnetic stimulation, stroke, upper extremity

## Abstract

*Background and Objectives:* Repetitive transcranial magnetic stimulation (rTMS) is being widely used for treating upper extremity paresis after stroke, however, evidence of applying high-frequency rTMS (HF-rTMS) on the ipsilesional hemisphere for upper extremity motor recovery remains limited. This systematic review aimed to investigate the effect of high-frequency repetitive transcranial magnetic stimulation for upper extremity motor function recovery after a first-time ischaemic stroke. *Materials and Methods:* This systematic review was prepared according to the preferred reporting items for systematic reviews and meta-analyses (PRISMA) guidelines. A comprehensive literature search was performed to identify all studies published before 12 February 2021. The search was performed on the following databases: PubMed, Ovid, The Cochrane Library. *Results:* A total of 6440 studies were found in the databases and four trials were included in the review. Three of the studies were randomized control trials (RCT), and one was a pseudo-RCT. Three of the studies showed good methodological quality and one study was rated as excellent. Fugl-Meyer Assessment (FMA) was performed in three out of four studies and the score significantly increased in the HF-rTMS treatment group compared with sham stimulation in all trials. Other measures used in the studies were handgrip strength, shoulder abduction, Motricity Index, Wolf Motor Function Test (WMFT), and Box and Block, although these tests did not show unanimous results. Overall, all four studies conveyed significantly better results in at least one test that was performed for hand motor function evaluation in a 10 Hz stimulation group while none of the tests showed any advantage for sham stimulation groups. Two studies reported headache as an adverse event (six patients in total). *Conclusion:* The overall results showed that HF-rTMS may increase impaired upper extremity motor function better than sham stimulation in stroke patients.

## 1. Introduction

Despite the growing knowledge about etiology and risk factors, stroke remains the leading cause of serious long-term adult disability worldwide [1,2]. Upper extremity paresis is one of the most common stroke residual effects since many patients fail to regain functional use of impaired arm [2,3,4]. Only half of the stroke survivors with plegic or paretic upper extremity regain useful hand movements within six months after stroke [5]. An improvement of this function has a positive significant effect on the quality of life of stroke patients, resulting in decreased economic and caregiver burdens [2].

A balance of function existing between the hemispheres, regulated by interhemispheric inhibition, is being affected after a stroke. The excitability of the contralesional hemisphere is increased hence the affected hemisphere undergoes an enhanced interhemispheric inhibition [6]. These excitability changes can be significant for impaired motor recovery of the affected extremity. Therefore, non-invasive neuromodulation technologies, such as repetitive transcranial magnetic stimulation (rTMS), have recently been started for use in the rehabilitation of stroke patients to improve upper extremity function [7]. It is believed that high-frequency rTMS (1 Hz and beyond—HF-rTMS) increases the cortical excitability in the affected hemisphere at the stimulation site [4] and improves the function of the affected extremity [8].

Repetitive transcranial magnetic stimulation is being widely used for treating upper extremity paresis after stroke [9,10]. However, the evidence of applying HF-rTMS on the ipsilesional hemisphere for upper extremity motor recovery remains limited [11,12,13]. The goal of this systematic literature review was to assess whether high-frequency rTMS delivered to the affected hemisphere motor cortex in stroke patients can improve upper extremity motor gains better than the sham stimulation.

## 2. Materials and Methods

### 2.1. Study Design

**Figure 1:**
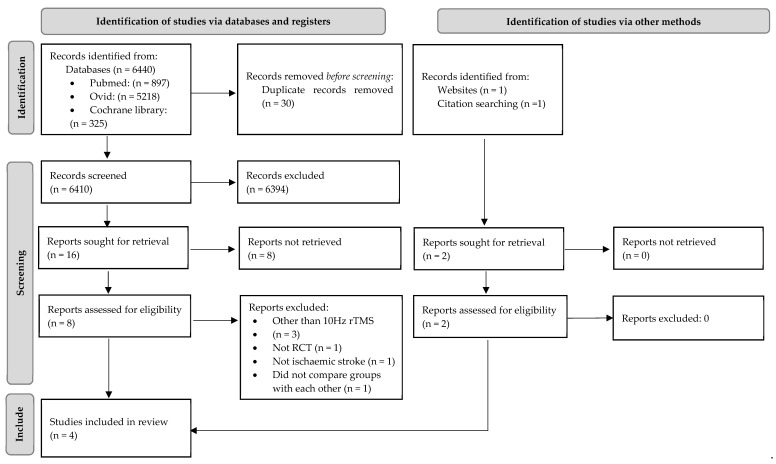
Prisma flow diagram for identification of studies.

This systematic review is prepared according to the preferred reporting items for systematic reviews and meta-analyses (PRISMA) guidelines. The study flow diagram is reported in Figure 1. The study was registered on the international prospective register of systematic reviews (PROSPERO) platform. Registration number: CRD42021229755.

### 2.2. Search Strategy

A comprehensive literature search was performed to identify all studies published before 12 February 2021. The search was performed on the following databases: PubMed, Ovid, and The Cochrane Library (Cochrane Central Register of Controlled Trials). The search strategies combined free text searching with keyword probing. Publications obtained from Medical Subject Heading (MeSH) were screened, using Boolean operators with the search functions “AND” and “OR”. The following query was used on PubMed: (rTMS OR TMS OR repetitive Transcranial Magnetic Stimulation OR “Transcranial Magnetic Stimulation” [Mesh]) AND (Stroke OR “Stroke” [Mesh]) AND (arm OR hand OR upper limb OR “Upper Extremity” [Mesh]). The specific search strategy is provided in Supplemental Digital Content 1 (https://1drv.ms/w/s!Ap1T2HUjzZkhgQY903jUdm7J_FxN?e=tSCg9x, accessed on 31 January 2021). Reference lists from the resulting publications were used to identify further relevant publications. Double-blind, randomized controlled trials (RCTs) or randomized, pseudo-RCTs studies were all included. Also, all articles uploaded to the Canadian Partnership for Stroke Recovery [14] website were surveyed for the same reason. Studies with more than two groups were included in the review if 10 Hz rTMS and sham stimulation groups were compared aside from each other. The authors did not contact any of the authors of the trials. Subsequently, each article was checked against the eligibility criteria.

### 2.3. Selection Criteria

Studies that met the following criteria were included in the present review: (1) double-blind RCTs, randomized, or pseudo-randomized controlled trials; (2) studies on adult patients (>18 years of age) with the first incidence of ischemic stroke and upper extremity hemiparesis; (3) where patients in the intervention group were administered high-frequency (10 Hz) rTMS alone or rTMS in combination with other treatments (physical therapy, occupational therapy, comprehensive rehabilitation therapy, and medications treatment), while participants in the control group were administered sham-rTMS or sham-rTMS in combination with other treatments mentioned above.

The exclusion criteria were as follows: (1) where the study method was not described; (2) studies reporting data on interventions different to 10 Hz frequency rTMS intervention; (3) studies of any other design than RCT (reviews, meta-analysis, case reports, or comments); (4) studies addressing another clinical problem (other than HF-rTMS effectiveness for upper extremity motor function recovery after first-time acute or subacute ischaemic stroke).

### 2.4. Data Extraction

All retrieved references were imported into the RefWorks ProQuest tool [15] and duplicates were removed. Titles and abstracts of retrieved studies were screened independently by two authors (B.V. and L.P.). Relevant full texts were assessed by the same authors who independently determined if they fulfilled the inclusion criteria. To achieve a consensus, a third reviewer was consulted when a difference in opinions arose. The following data was obtained from each of the included studies: (1) study details: authorship and publication date; (2) sample characteristics: mean age and sex and the type and stage of stroke; (3) description of interventions: frequency, stimulation site, intensity, pulses per session, number of treatment sessions, and total sessions; (4) follow-up period; (5) upper extremity motor function assessment (scales and tests); (6) hand motor function outcome measures.

### 2.5. Quality Assessment

The quality of selected studies was analysed using The Physiotherapy Evidence Database (PEDro) rating scale [16]. Two independent reviewers evaluated whether each quality criterion was fulfilled (score 1) or not (score 0) and counted the total score for each study. To achieve a consensus, the third reviewer was consulted when the difference in opinions arose. The methodological quality of the study was considered poor when the final score for the article was three or below, fair when the score was four or five, good when the score ranged from six to eight, and excellent when the score reached nine or ten.

## 3. Results

### 3.1. Study Selection

A total of 6440 studies were found in databases. A total of 30 records were excluded as duplicates, 6394 records were excluded based on their titles and abstracts, and 8 records were not retrieved. The remaining 16 studies were assessed for eligibility. A total of 3 studies were excluded because a frequency other than 10 Hz rTMS was used [17,18,19], one study was excluded because included patients experienced haemorrhagic and non-ischemic stroke [20], one study was not RCT [21], and one study did not compare rTMS and sham stimulation groups with each other [22]. Two articles were identified via other methods: one was found during citation searching [23] and one was found on a website [19]. In total, four trials were included in the systematic review [23,24,25,26]. To minimize the risk of bias, two authors independently reviewed the titles, abstracts, and full text articles.

### 3.2. Study Characteristics

The study characteristics are presented in Table 1. The studies spanned from 2009 to 2017. Three of the studies were randomised control trials (RCT) [23,24,26], and one was a pseudo-RCT [25]. The randomisation of subjects in the latter study was performed using the table of random sampling numbers. When the last number showed a multiple of three, subjects were allocated into the sham stimulation group, and the other subjects were designated into the real rTMS group. One study examined individuals with acute stroke [23] and three studies examined individuals with subacute stroke [24,25,26]. According to the PEDro rating scale, three of the studies showed good methodological quality [23,25,26] and one study was rated as excellent [24], (Table 1).

### 3.3. Outcomes

The Fugl-Meyer Assessment (FMA) was performed in three out of four studies to evaluate the efficacy of high-frequency rTMS on upper extremity motor function recovery. In all studies, FMA scores significantly increased in the HF-rTMS treatment group compared with sham stimulation. Handgrip was tested in two trials. In one of them, handgrip was significantly better in HF-rTMS group. The second study reported an equal increase in muscle strength in both groups. Other tests used in the studies did not show unanimous results. The shoulder abduction and Motricity Index improved significantly in HF-rTMS groups over the Wolf Motor Function Test (WMFT). Box and Block test (BBT) did not show any differences between groups.

In two studies no adverse events were reported [23,25]. In one study, four patients withdrew from the trial because they were unable to tolerate the pain caused by HF-rTMS, [24] and one study reported two subjects experiencing transient headaches at the beginning of stimulation (both in HF-rTMS group) [26].

## 4. Discussion

To evaluate the efficacy of high-frequency (10 Hz) rTMS for upper extremity motor function recovery after ischemic stroke, FMA-UL, WMFT, MI-A, BBT, the strength of handgrip, and shoulder abduction testing were performed in included trials [23,24,25,26]. We found that FMA-UL scores in HF-rTMS groups increased significantly more than in controls. Shoulder abduction and Motricity Index arm score (MI-A) also showed significantly better scores in rTMS group participants. The strength of the handgrip was tested in two trials and the outcomes differed. One study provided handgrip evaluation results in favour of the stimulation group, and another did not find any differences between groups. In one study, motor function was assessed using WMFT; however, this did not show any significant differences between the rTMS and control groups. Additionally, the same result was observed in BBT performance. Despite the fact that both FMA and WMFT are stroke-specific tests, FMA is proven to have better predictive validity and is significantly more responsive than WMFT. This might be explained by the fact that FMA covers a wider range of assessment in the upper extremity function rather than WMFT, which is limited to the measures of gross motion [27]. Overall, all four studies conveyed significantly better results in at least one test that was performed for hand motor function evaluation in a 10 Hz stimulation group while none of the tests showed any advantage for sham stimulation groups.

No significant adverse events, related to stimulation, were reported in the studies. However, a total of six individuals experienced headaches, while four of them were unable to tolerate the pain and dropped out of the research. Nevertheless, no further harm was reported, suggesting that rTMS is a safe procedure for upper extremity motor recovery after ischemic stroke. It is well known that rTMS procedures might provoke adverse effects such as seizures, headaches, local pain, neck pain, and transient hearing changes. Headache is the most common adverse event of rTMS and it is believed to occur more often if a higher frequency is being used [28,29]. Possible explanations could be the direct stimulation of superficial nerves and muscles which depend on coil position or the increased cerebral blood flow as a response to stimulation, or both [30]. However, one recent systematic review reported 28 adverse effects which were minor and transient but none of them occurred in the HF-rTMS group [31]. As mentioned above, in our systematic review, headache was the only reported side effect; however, it accounted for a small number of patients (six patients in total). Headache was reported in both studies that used a total number of pulses higher than 1000 during one session; however, it is beside the purpose to jump to conclusions, since both mentioned studies included a bigger number of participants in their trials, which simply might have given a better probability for adverse events to occur. Although rTMS is considered a safe procedure for stroke patients, researchers should always follow safety recommendations and evaluate the risk ratio individually, especially for those who have a higher risk of seizure [28].

Only studies with a sham stimulation control group were included in the review to rule out the placebo effect. Due to few RCTs found after the screening one pseudo-RCT was included. The referred trial was evaluated the same way as other studies using the PEDro rating scale. The main disparity from the RCTs was that the person who distributed subjects into groups was aware of which group the participant was allocated to. Despite this fact, it is unlikely to have an effect on the final results, since pseudo-RCT showed good methodological quality.

Currently, the rTMS procedure protocols are poorly defined; therefore, some significant differences were observed in the design. It is believed that different stimulation parameters might have an impact on both overall results and adverse events. Moreover, various examination methods were used to evaluate hand motor function. This important aspect could have had a significant impact on overall results too. The time of assessment was another relevant difference in the designs of the studies. To make trials more homogenous, only pre- and post-treatment assessment results comprised the review.

In three of four studies, a small number of patients (less than 20 patients) were included in the HF-rTMS group [23,25,26], which could have affected the final results. A common recommendation would be to include more participants in future studies for homogenous results.

## 5. Conclusions

Despite differences in designs and outcome measures and the limitations of small sample sizes in trials, the overall results showed that HF-rTMS may increase impaired upper extremity motor function more successfully than sham stimulation in stroke patients. A more suitable statistical estimation of our question could be resolved by a meta-analysis; however, the number of eligible studies is too low at this stage. Further studies, with larger, randomized, controlled samples, are needed to better estimate the efficacy and safety of HF-rTMS for upper extremity motor function recovery in stroke patients.

## Figures and Tables

**Table 1:** Study characteristics of the studies included in the systemic review, examining the effect of HF-rMTS for upper extremity motor recovery after first-time ischemic stroke.

Trial Design/ Quality	Regimen	Age (Years)	M/F	10 Hz-rTMS Group Frequency And Intensity	No. of rTMS Sessions	No of Pulses/Session	Outcome Measures	Main Findings	Time of Assessment	Adverse Event
RCT/6	C: Sham (*n* = 42) E1: 1 Hz over non-affected hemisphere (*n* = 42) E2: 10 Hz over affected hemisphere (*n* = 43)	53.13 ± 13.72 57.87 ± 12.89 54.00 ± 13.35	28/14 30/12 29/14	10 Hz 80% RMT	5 days/week, 2 weeks (10 sessions)	C: =E2 E1: 1000 pulses (10 s stimulation, 2 s rest); E2: 1350 pulses (1.5 stimulation, 10 s rest);	FMA, WMFT	FMA (+E2) WMFT (NS)	Baseline, after treatment	Four patients were unable to tolerate the pain caused by stimulation (withdrawn from the trial)
RCT/8	C: Sham (*n* = 16) E1: 3 Hz (*n* = 16) E2: 10 Hz (*n* = 16)	58 ± 11.64 58.25 ± 15.07 58.37 ± 13.96	9/7 8/8 7/9	10 Hz 100% RMT	5 days/week, 1 week (5 sessions)	C: 750 pulses (5 s, 50 train); E1: 750 pulses (2 s, 37 train); E2: 750 pulses (2 s, 37 train);	handgrip, shoulder abduction	Strength of hand grip (NS) Shoulder abduction (+E2)	Baseline, after treatment, month 1, 2, 3; after 1 year	no adverse effect
pseudo- RCT/6	C: Sham (*n* = 10) E: 10 Hz (*n* = 18)	57.0 ± 14.5 56.4 ± 11.2	6/4 11/7	10 Hz 90% RMT	5 days/week, 2 weeks (10 sessions)	C: 1000 pulses (5 s, 50 train) E: 1000 pulses (5 s, 50 train)	MI-A, FMA-UL, BBT, grip strength	MI-A (+E) FMA-UL (+E) Grip strength (+E) BBT (NS)	Baseline, after treatment, after 3 months	no adverse effect
RCT/10	C: Sham (*n* = 20) E1: 10 Hz (*n* = 20) E2: 1 Hz (*n* = 20)	56 ± 11 (35–72) 54 ± 12 (30–68) 56 ± 9 (32–69)	16/4 14/6 18/2	10 Hz 100% RMT	5 days/week, 2 weeks (10 sessions)	C: =E2 E1: 1200 pulses (4 s, 40 s interval between session); E2: 1200 pulses (120 s, 40 s interval between session);	FMA	FMA (+E)	Baseline, after treatment, after 3 months	two transient headaches at the beginning of stimulation

RCT—randomized clinical trial; FMA-UL—Fugl-Meyer Assessment scale for upper limb; WMFT—Wolf Motor Function Test; MI-A—Motricity Index arm score; BBT—Box and Block Test; MRC—Medical Research Council; NS—not significant.

## Data Availability

Not applicable.
