# Correction: Vabalaite et al. Effects of High-Frequency (HF) Repetitive Transcranial Magnetic Stimulation (rTMS) on Upper Extremity Motor Function in Stroke Patients: A Systematic Review. *Medicina* 2021, *57*, 1215

**DOI:** 10.3390/medicina58040533

**Published:** 2022-04-12

**Authors:** Birute Vabalaite, Laura Petruseviciene, Raimondas Savickas, Raimondas Kubilius, Povilas Ignatavicius, Egle Lendraitiene

**Affiliations:** 1 Department of Rehabilitation, Hospital of Lithuanian University of Health Sciences Kauno Klinikos, Eiveniu Str. 2, LT-50161 Kaunas, Lithuania; lciginskaite@gmail.com (L.P.); raimondas.savickas@kaunoklinikos.lt (R.S.); raimondas.kubilius@kaunoklinikos.lt (R.K.); egle.lendraitiene@kaunoklinikos.lt (E.L.); 2 Department of Rehabilitation, Lithuanian University of Health Sciences, Mickeviciaus Str. 7, LT-44307 Kaunas, Lithuania; 3 Department of Surgery, Hospital of Lithuanian University of Health Sciences Kaunas Klinikos, Eiveniu Str. 2, LT-50161 Kaunas, Lithuania; povilas.ignatavicius@kaunoklinikos.lt

In the original publication [1], there was an error regarding the Affiliations for Birute Vabalaite, Laura Petruseviciene, Raimondas Savickas, Raimondas Kubilius and Egle Lendraitiene. In addition to Affiliation 1, Affiliation 2 should have been newly added. The previous Affiliation 2 has been changed to Affiliation 3. The corrected Affiliations are as follows:

^1^  Department of Rehabilitation, Hospital of Lithuanian University of Health Sciences Kauno Klinikos, Eiveniu Str. 2, LT-50161 Kaunas, Lithuania; lciginskaite@gmail.com (L.P.); raimondas.savickas@kaunoklinikos.lt (R.S.); raimondas.kubilius@kaunoklinikos.lt (R.K.); egle.lendraitiene@kaunoklinikos.lt (E.L.)
^2^  Department of Rehabilitation, Lithuanian University of Health Sciences, Mickeviciaus Str. 7, LT-44307 Kaunas, Lithuania
^3^  Department of Surgery, Hospital of Lithuanian University of Health Sciences Kaunas Klinikos, Eiveniu Str. 2, LT-50161 Kaunas, Lithuania; povilas.ignatavicius@kaunoklinikos.lt

The authors apologize for any inconvenience caused, and state that the scientific conclusions are unaffected. This correction has been approved by the Academic Editor. The original publication has also been updated.
